# Supplementary material for: Dietary Long-Chain n-3 Polyunsaturated Fatty Acid Supplementation Alters Electrophysiological Properties in the Nucleus Accumbens and Emotional Behavior in Naïve and Chronically Stressed Mice
Source: Int J Mol Sci. 2022 Jun 14;23(12):6650. doi: 10.3390/ijms23126650 (PMC9224532; doi:10.3390/ijms23126650)
Supplement: Supplementary file 1 [file ijms-23-06650-s001.zip › IJMS 1744977 Supplemental materials.pdf]

Table S1: Statistical tests performed in the present study. n/a: non-applicable, ANOVA: analysis of variation, RM: repeated measures, p values are encoded as following: * p<0.05, ** p<0.01 and *** p<0.001.

Table S2: Genes used for mRNA microarray. Gene names, aliases and functions are presented.

Gene name	Full name	Other name(s) in *mus musculus*	Protein function
Alox5	Arachidonate 5-lipoxygenase	5-LO, 5-LOX, 5LO, 5LX, AI850497, F730011J02	Lipoxygenase
Alox8	Arachidonate 8-lipoxygenase	15-LOX-2, 15-LOX-B, 8-LOX, 8S-LOX, Alox15b	
Alox15	Arachidonate 15-lipoxygenase	12-LOX, 15-LOX, 15-LOX-1, LOG15	
Abhd4	Abhydrolase domain containing 4	1110035H23Rik, AI429574, Abh4	Hydrolase
Abhd6	Abhydrolase domain containing 6	0610041D24Rik, AA673485, AV065425	
Abhd12	Abhydrolase domain containing 12	1500011G07Rik, 6330583M11Rik, AI431047, AW547313	
Acaa1a	Acetyl-Coenzyme A acyltransferase 1A	Acaa, Acaa1, D9Ertd25e, PTL	Transferase
Acaca	Acetyl-Coenzyme A carboxylase alpha	A530025K05Rik, Acac, Acc1, Gm738	Carboxylase
Acadl	Acyl-Coenzyme A dehydrogenase, long-chain	AA960361, AU018452, C79855, LCAD	Dehydrogenase
Acadvl	Acyl-Coenzyme A dehydrogenase, very long chain	vlcad	
Acat1	Acetyl-Coenzyme A acetyltransferase 1	6330585C21Rik, Acat	Transferase
Acox1	Acyl-Coenzyme A oxidase 1	AOX, Acox, D130055E20Rik, Paox	Oxidase
Acsl1	Acyl-CoA synthetase long-chain family member 1	Acas, Acas1, Acs, FACS, Facl2, LACS 1, LACS1	Ligase
Actb	Actin, beta	Actx, E430023M04Rik, beta-actin	Housekeeping
Apidor1	Adiponectin receptor 1	2810031L11Rik, ACDCR1, CGI-45, Paqr1	Receptor
Adipor2	Adiponectin receptor 2	1110001I14Rik, ADCR2, AI115388, AW554121, D6Ucla1e, Paqr2	
Apoe	Apolipoprotein E	AI255918, Apo-E	Transporter
Cnr1	Cannabinoid receptor 1	CB1, CB-R, CB1A, CB1B, CB1R	Receptor
Cnr2	Cannabinoid receptor 2	CB2, CB-2, CB2-R	
Cd36	CD36 molecule	FAT, GPIV, Scarb3	Protein
Cmklr1	Chemokine-like receptor 1	ChemR23, DEZ, Gpcr27, mcmklr1	Receptor
Cpla2	Phospholipase A2, group IVA	Pla2g4, cPLA2, cPLA2-alpha, cPLA2alpha	Lipase
Cs	Citrate synthase	2610511A05Rik, 9030605P22Rik, Ahl4, BB234005, Cis	Synthase
Cyp27a1	Cytochrome P450, family 27, subfamily a, polypeptide 1	1300013A03Rik, Cyp27	Monooxygenase
Dagla	Diacylglycerol lipase, alpha	Nsddr	Lipase
Elovl1	Elongation of very long chain fatty acids-like 1	Ssc1, AA407424, BB151133	Elongase
Elovl2	Elongation of very long chain fatty acids-like 2	Ssc2, AI317360	
Elovl4	Elongation of very long chain fatty acids-like 4		
Elovl5	ELOVL family member 5	HELO1, AI747313, AU043003, 1110059L23Rik	
Enpp2	Diacylglycerol lipase, alpha	Nsddr	Lipase
Faah	Fatty acid amide hydrolase	AW412498	Hydrolase
Fapb7	Fatty acid binding protein 7	MRG, Blbp, BFABP, B-FABP	Binder
Fads1	Fatty acid desaturase 1	DSD, AI317215, 0710001O03Rik, A930006B21Rik	Desaturase
Fads2	Fatty acid desaturase 2	Fads2, Fadsd2, 2900042M13Rik	
Fasn	Fatty acid synthase	FAS, A630082H08Rik	Synthase
Fpr2	Formyl peptide receptor 2	E330010I07Rik, Fpr-rs2	Receptor
Gapdh	Glyceraldehyde-3-phosphate dehydrogenase	Gapd	Dehydrogenase
Gnpat	Glyceronephosphate O-acyltransferase	AU019525, D1Ertd819e, DHAPAT	Transferase
Gpr18	G protein-coupled receptor 18		Receptor

Gpr31b	G protein-coupled receptor 31	12-HETER, GPR31c(t), Gpr31, Gpr31c	
Gpr55	G protein-coupled receptor 55	CTLF, Gm218, Lpir1	
Hadh	Hydroxyacyl-Coenzyme A dehydrogenase	AA409008, AU019341, AW742602, HCDHsc, Schad	Dehydrogenase
Hsd17b4	Hydroxysteroid 17-beta dehydrogenase 4		
Ipla2	Phospholipase A2, group VI	iPLA2, PNPLA9, BB112799, iPLA2beta, iPLA(2)beta	Lipase
Ltc4s	Leukotriene C4 synthase		Synthase
Magl	Monoglyceride lipase	Mgl, Magl, AA589436	Lipase
Napepld	N-acyl phosphatidylethanolamine phospholipase D	Mbldc1, NAPE-PLD, A530089G06	
Nat1	N-acetyltransferase 1	Nat-1	Transferase
Nat2	N-acetyltransferase 2	Nat2a	
Pcx	Pyruvate carboxylase	Pc, Pcb	Carboxylase
Pcyt1a	Phosphate cytidylyltransferase 1, choline, alpha isoform	CTalpha, Cctalpha, Ctpct, Cttalpha	Transferase
Pcyt1b	Phosphate cytidylyltransferase 1, choline, beta isoform	AW045697, CTTbeta	
Pcyt2	Phosphate cytidylyltransferase 2, ethanolamine	1110033E03Rik, ET	
Pla1a	Phospholipase A1 member A	Pspla1, Ps-pla1, AA986889	Lipase
Plcb1	Phospholipase C, beta 1	Plcb, AI132408, mKIAA0581, 3110043I21Rik	
Plcb2	Phospholipase C, beta 2	AI550384, B230399N12, B230205M18Rik	
Plcb3	Phospholipase C, beta 3	mKIAA4098	
Plpp1	Phospholipid phosphatase 1	LPP1, mPAP, Hic53, LPP-1, PAP2a, Hpic53, PAP-2a, Ppap2a, PAP2-alpha	Phosphatase
Ppara	Peroxisome proliferator activated receptor alpha	4933429D07Rik, AW742785, Nr1c1, PPAR-alpha, PPARalpha, Ppar	Receptor
Ppard	Peroxisome proliferator activated receptor delta	NUC-1, NUC1, Nr1c2, PPAR-beta, PPAR-delta, PPAR[b], PPARdelta, Pparb, Pparb/d	
Ppargc1a	Peroxisome proliferative activated receptor, gamma, coactivator 1 alpha	A830037N07Rik, Gm11133, PGC-1, PPARGC-1-alpha, Pgc-1alpha, Pgc1, Pgco1, Ppargc1	
Ppargc1b	Peroxisome proliferative activated receptor, gamma, coactivator 1 beta	4631412G21Rik, PGC-1beta, PGC-1beta/ERRL1, PPARGC-1-beta, Perc	
Pparg	Peroxisome proliferator activated receptor gamma	Nr1c3, PPAR-gamma, PPAR-gamma2, PPARgamma, PPARgamma2	
Ptafr	Platelet-activating factor receptor	PAFR	
Ptgd2	Hematopoietic prostaglandin D synthase	Hpgds	Synthase
Ptger1	Prostaglandin E receptor 1	EP1, Ptgerep1	Receptor
Ptger3	Prostaglandin E receptor 3	EP3, Pgerep3, Ptgerep3	
Ptger4	Prostaglandin E receptor 4	EP4, Ptger, Ptgerep4	
Ptges	Prostaglandin E synthase	2410099E23Rik, D2Ertd369e, Pges, mPGES, mPGES-1	Synthase
Ptgfr	Prostaglandin F receptor	AI957154, PGF, fp	Receptor
Ptgis	Prostaglandin I2 (prostacyclin) synthase	Cyp8, Cyp8a1, Pgis	Synthase
Ptgs1	Prostaglandin-endoperoxide synthase 1	COX1, Cox-1, Cox-3, PGHS-1, PHS 1, Pghs1	
Ptgs2	Prostaglandin-endoperoxide synthase 2	COX2, Cox-2, PES-2, PGHS-2, PHS II, PHS-2, Pghs2, TIS10, gripghs	
Rara	Retinoic acid receptor, alpha	Nr1b1, RAR, RARalpha1	Receptor
RnaseP	Ribonuclease P		Nuclease
Rxra	Retinoid X receptor alpha	9530071D11Rik, Nr2b1, RXRalpha1	Receptor
Rxrb	Retinoid X receptor beta	AL023085, H-2RIIBP, Nr2b2, RCoR-1, Rub	
Rxrg	Retinoid X receptor gamma	Nr2b3	
Scd1	Stearoyl-Coenzyme A desaturase 1	AA589638, AI265570, Scd, Scd-1, ab	Desaturase
Sirt1	Sirtuin 1	AA673258, SIR2L1, Sir2, Sir2a, Sir2alpha	Regulator

Slc2a1	Solute carrier family 2, member 1	Glut1, Glut-1, M100200, Rgsc200	
Slc2a3	Solute carrier family 2, member 3	Glut3, C78366, Glut-3, AA408729, AL023014, AL024341, AU040424	Transporter
Slc2a5	Solute carrier family 2, member 5	Glut5, Slc5a, AI526984	
Slc25a20	Solute carrier family 25, member 20	CAC, Cact, mCAC, C78826, 1110007P09Rik	
Slc27a1	Solute carrier family 27, member 1	Fatp, FATP1	Carrier
Slc27a3	Solute carrier family 27, member 3	FATP3, Acsvl3, FATP-3, Vlcs-3	
Slc27a4	Solute carrier family 27, member 4	FATP4, ACSVL4, BB144259	
Tbax2r	Thromboxane A2 receptor	TP, TXAR-2	Receptor
Tbxas1	Thromboxane A synthase 1	CYP5, TS, TXS, THAS, CYP5A1	Synthase

Table S3: Gene names and forward/reverse primer sequences used in the present study.

Gene	Forward primer sequence	Reverse primer sequence
Alox5	TTCCCATTGCCATCCAGCTC	TGGAAGTCACTGGAACGCAC
Alox8	TGGCCACATTACGTCAGCTG	GCGTGTTGATGTGCAGTGTG
Alox15	GCTGTTGCCACCATGAGATG	AACGGATGTGTGGAACGAGG
Abhd4	TTCCGTCCAGACTTCAAGCG	GCTTTGAATGCCGTTTCCCC
Abhd6	TTGCGTCTTTCCTCCTGTGG	AACTTGCATGCCCAGTGTCC
Abhd12	TGTCTGGTGGAAGAATGCCC	TTGTACAGCTCCACACGGTG
Acaa1a	AAAGCCAGAGACTGCCTGAC	CTGCCGTGAAATGCCAAACC
Acaca	ATGACGGCAGCAGTTACACC	AGACGGTGAGCGCATTACAG
Acadl	GCTGAGTTGGCGATTTCTGC	TGCTGCACCGTCTGTATGTG
Acadvl	TTTGGCCTGCAAGTACCCAG	TCTGCCAAGCGAGCATACTG
Acat1	TCAGTGTGGTTGTGCTAGCC	AACAACAATCCGGGCTCCAG
Acox1	TCACTCGAAGCCAGCGTTAC	TTGAGGCCAACAGGTTCCAC
Acsl1	AAACTTGGGAAGGAAGCCGG	TTGGAGTCAGAAGGCCGTTG
Actb	TACAATGAGCTGCGTGTGGC	ACATGGCTGGGGTGTTGAAG
Apidor1	ATTCCTGAGCGCTTCTTCCC	GAAGTGGACGAAAGCTGCTG
Adipor2	ATCCCTGAGCGCTTCTTTCC	TGCAGGTTTGAGACTCCGTG
Apoe	TGCGAAGATGAAGGCTCTGTG	GGTTGGTTGCTTTGCCACTC
Cnr1	CCTGGGAAGTGTCATCTTTGTCT	GGTAACCCCACCCAGTTTGA
Cnr2	AAACAATGCTCTCCAGGGCC	TGGGAGCCAGAAGTCACATG
Cd36	TCCCTTGATTCTGCTGCACG	AGCCAGGACTGCACCAATAAC
Cmklr1	AGTCACGCGCAGTAACAGAC	TCGTTGTAAGCGTCGTACTCC
Cpla2	AAAGTACAAGGCCCCAGGTG	AATGGCGATTCGGGGTCATC
Cs	ACTCAATTCAGGACGGGTGG	AGCAAACTCTCGCTGACAGG
Cyp27a1	TCGGAGGATTGCAGAACTGG	ATGCAGCCTCACCTTCTTGC
Dagla	TCTGCGGACTTACAACCTGC	ACACTTTTAGACGGCGGGAC
Elovl1	AAGAAGGACGGGCAAGTGAC	ATCATGGCATGGAAGGAGCC
Elovl2	ATGTCTATCACCACGCGTCC	ACTTGTGCATGGACGGGAAC
Elovl4	AAACGTGTAGCAGACTGGCC	CTAAGCGCATTTGGAACGGC
Elovl5	TACATGAAGAACCGGCAGCC	TTTGCCTTCCCACACACCTG
Enpp2	AGCATTCAGGGCAAGCAGAG	AGCAGAGATTGACGCCGATG
Faah	CTTTGTGCACACCAACGTCC	TTCCACGGGTTCATGGTCTG
Fapb7	AGGAAGGTGGCAAAGTGGTG	AGCTTGTCTCCATCCAACCG
Fads1	GCAACGTTCACCAATCAGCC	TACTTGGCGCACAGGGATTG
Fads2	GCATGTGTTTGTCCTTGGCG	TCATGCTGGTGGTTGTAGGG
Fasn	CCAAGCGGCCATTTCCATTG	TGTCCCTCGAGTTGGCAAAG
Fpr2	GGACCGCTGCATTTGTGTTC	AAATCCAGGGCCCAACAACC
Gapdh	TGAACGGATTTGGCCGTATTG	CGTTGAATTTGCCGTGAGTG
Gnpat	TCACTGCGTTCTCTAGCTGC	AAAGGCTTGCACGTCCTCAG
Gpr18	GCGTTGTGGGTTTTCAGCTG	ACGAGGTCCAGTAGTGCAAC
Gpr31b	AGTGTGTGCTGGGCCTTATG	GCAGCAAAGAATGGCAGACTG
Gpr55	TATTCACATCCTGCTGCGCC	AATGGTCCAGATGCAGGCTC
Hadh	AAACACCGATGACCAGCCAG	ATGGCACCAAGAGTCGGTTC
Hsd17b4	CTTCAGCAATGCCAGCAAGC	AGCACCAACGAATCCTGACG

Ipla2	ACTCCATTGGGCCAAGAACG	AGCAGCACCATGACACAGTC
Ltc4s	TCTCTGCACGAAGGGCTTTC	TTCGTGGAAGAAGATGCCGG
Magl	TGCTGTCTCGGAACAAGTCG	ATTGCTCGCTCCACTCTTGC
Napepld	ACTGCCCCGCTTTTGAAGAG	TCTTCTGGGTCTGCATGCTG
Nat1	ATCATCTGCTGTACTGGGCTC	TTCCTGTCACTGATGGTCACC
Nat2	GTTGATGCTGGGTTTGGACG	TCAAACGGAAGATGGCAGGC
Pcx	TCCGGTTCATTGGTCCAAGC	TTGGAGAACTCATGCGCCTC
Pcyt1a	CCGGATTGATTTTGTCGCCC	CAAACATGCCTGCGTCCTTG
Pcyt1b	TGGCTTCTGACATGCTGTGG	ACAACCCACCAGTGTAACCG
Pcyt2	TTTGCTTCTGGGAAGGAGCC	TGGAACAGGTCAAAGGCACC
Pla1a	TGGATGCCCTGCCTTCTTTC	ATCAGTGGGCAGGTGTTCTC
Plcb1	AGTAGAGGCGCAAACCATCG	TGTACTTGGTCGTGTGCTCC
Plcb2	TCCTGCTGATCGAAAACGGG	ATCTCGTCGATTTCTGGCCG
Plcb3	CAACCAACCTTTGTGCCTGC	AGGCTTACGTGCTTGATGGG
Plpp1	AAGTCAAGGAGGGCAGGTTG	GCGACAAACAGCATGCAGTAC
Ppara	ATGAACAAGGTCAAGGCCCG	AAGCGTCTTCTCGGCCATAC
Ppard	ACGCACCCTTTGTCATCCAC	TCACCAGCTGTTTCCACACC
Ppargc1a	ACTACAGACACCGCACACAC	AGCCTTTCGTGCTCATAGGC
Ppargc1b	TCAGTTCCAGAAGTCAGCGG	ATGCAGTTCCGTACAAGGCC
Pparg	TCAGAAGTGCCTTGCTGTGG	ACAGCTTCTCCTTCTCGGC
Ptafr	TCATCATCCACACGCTGCTC	TGAATACCGCCAAGACCGTG
Ptgd2	TGCTGTGGATGGGTTTGGTC	GAAAGTTGGGCTGCACTGTG
Ptger1	TGCCACCTTCCTGTTGTTCG	AAGAAGACCATGCAGCCACC
Ptger3	ACCATCAAAGCCCTGGTGTC	AGACGGACAGCACACACATG
Ptger4	ATCGAACCGTGAGCTCCAAC	ATCACTGCGGGAATGGTCAC
Ptges	ATCAAGATGTACGCGGTGGC	ATCCTCGGGGTTGGCAAAAG
Ptgfr	TCTTCTGCTCCGGACACAAC	AGACACTGGCTGCTTGGAAC
Ptgis	TGCCTTGGAGTTTGGGAGAG	AACAGTGACGTATCTGCCCC
Ptgs1	ATCACCTGCGGCTCTTCAAG	ATCAACACGGACGCCTGTTC
Ptgs2	AGGAACTCAGCACTGCATCC	TCCCCACGGTTTTGACATGG
Rara	AATGTTTCGACGTGGGCATG	TTCTCAATGAGCTCGCCCAC
Rxra	TACGTGGAGGCAAACATGGG	AGGGGCAGCTCAGAAAAGTG
Rxrb	ACGGGTCTTCATGTGCACAG	TGTCCATCCTCATGTCACGC
Rxrg	AGTAGCCACGAAGACATGCC	TCTCCACGTTCATGTCACCG
Scd1	AGGGCGGAAAACTGGACATG	TACAAAAGTCTCGCCCCAGC
Sirt1	TCATGGTTCCTTTGCAACAGC	GGCTTCATGATGGCAAGTGG
Slc2a1	TGCTGTGCTCATGACCATCG	AAGATGGCCACGATGCTCAG
Slc2a3	TGGCTGGCTGTTGTAACTGG	AGATGAGGAAGGCAGCGAAG
Slc2a5	TGATTGTCCTCATGGCTGGC	ATGAACACATTGACGGCCCC
Slc25a20	ATGCGAGATGTTCCTGCCAG	AAGATCCCTGCAAAGCCACC
Slc27a1	TGTGCTCTATGACTGCCTGC	ACTTCTTGCGCAGTACCACC
Slc27a3	AGAACTTGCCACCGTATGCC	TGGCCATCCTAACCTTCTGC
Slc27a4	AGTACATTGGCGAGCTCTGC	TGTGGAAACGGCTGGAGAAG
Tbax2r	TGCTCATCTACCTGCGTGTG	TGGAGCTGTGAACTGAACCG
Tbxas1	TGCCGTATCTGGACATGGTG	TCCCGTGTGAACCTGAAAGC

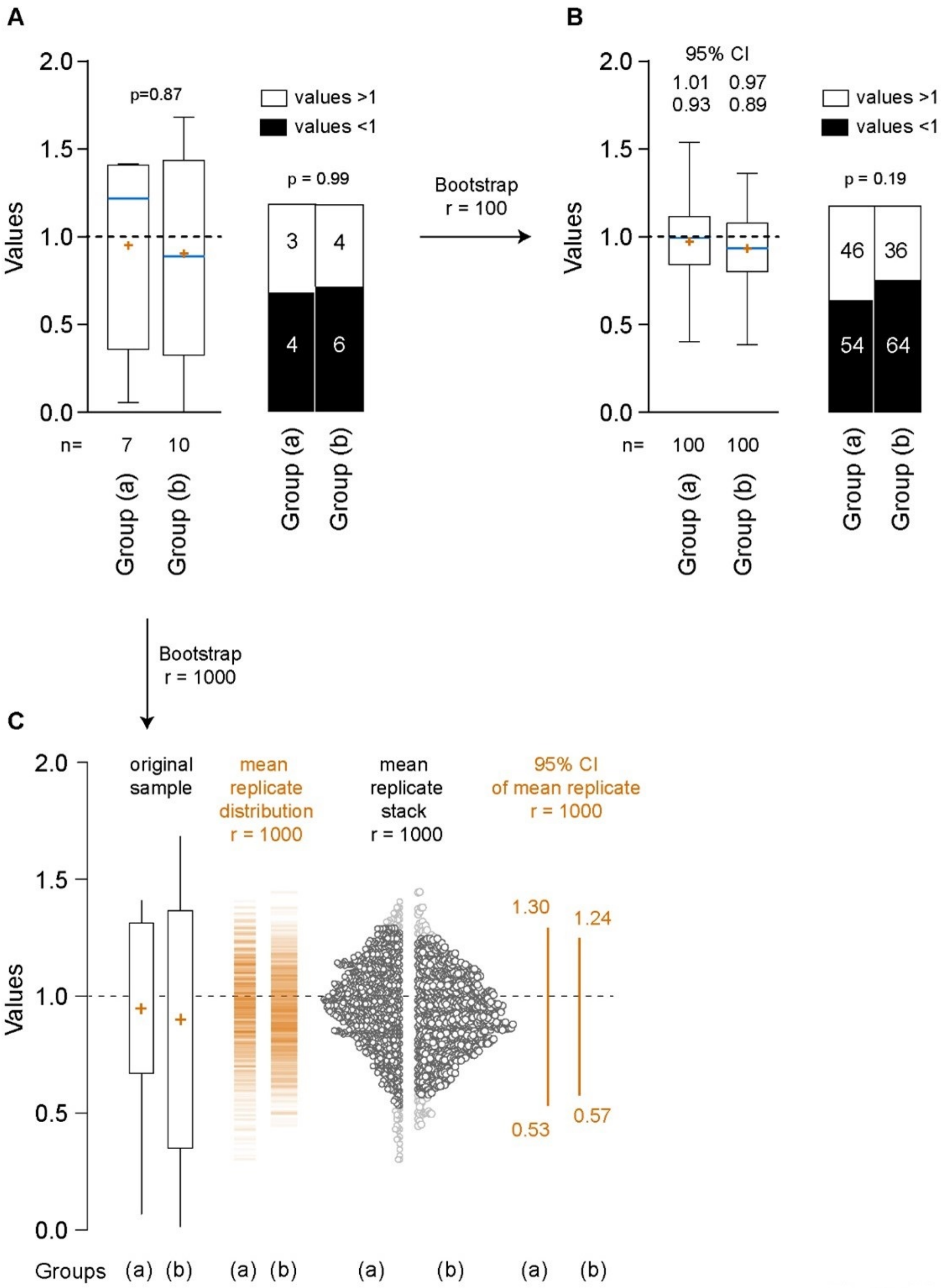

Figure S1. Bootstrap resampling of group mean. (**A**) Based on our previous results obtained in Figure 4A, we generated bootstrapped values, using input parameters matched to the in vivo measurements (identical means and standard deviations). Subsequent random sampling with 7 and 10 measures were then performed to yield newly-generated samples (n=7 and n=10 in groups a and b, respectively). These generated values presented similar distributions than what was observed before (Figure 4A), thus validating the present resampling technique. Note that the distribution of values below (<1) or above (>1) threshold are not significantly different in the two groups (a and b, p=0.99). (**B**) We controlled and confirmed that such a resampling technique with 100 random measurements (r=100) does not reach statistical significance *per se*. Note how mean values (orange crosses) are conserved throughout the resampling. 95% CI: 95% confidence intervals. Orange crosses represent group means while horizontal blue lines represent group median values. (**C**) Using the visual inference tool (VIT) from iNZight [87,88], we then ran extreme resampling (r=1000) of the dataset presented in **A**. As expected, such a method produced greatly overlapping mean distributions (orange crosses and horizontal orange lines) and 95% CI. Faded circles are values outside of the 95% CI.

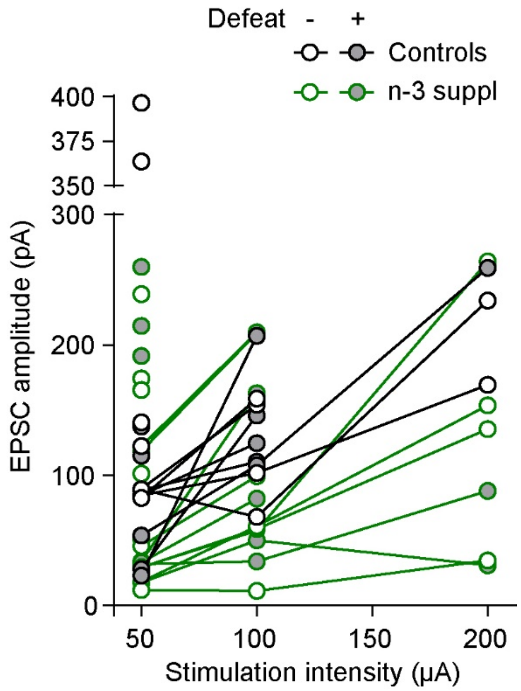

Figure S2. Input-output (stimulation intensity in relation to EPSC amplitude) relationships in the different groups.

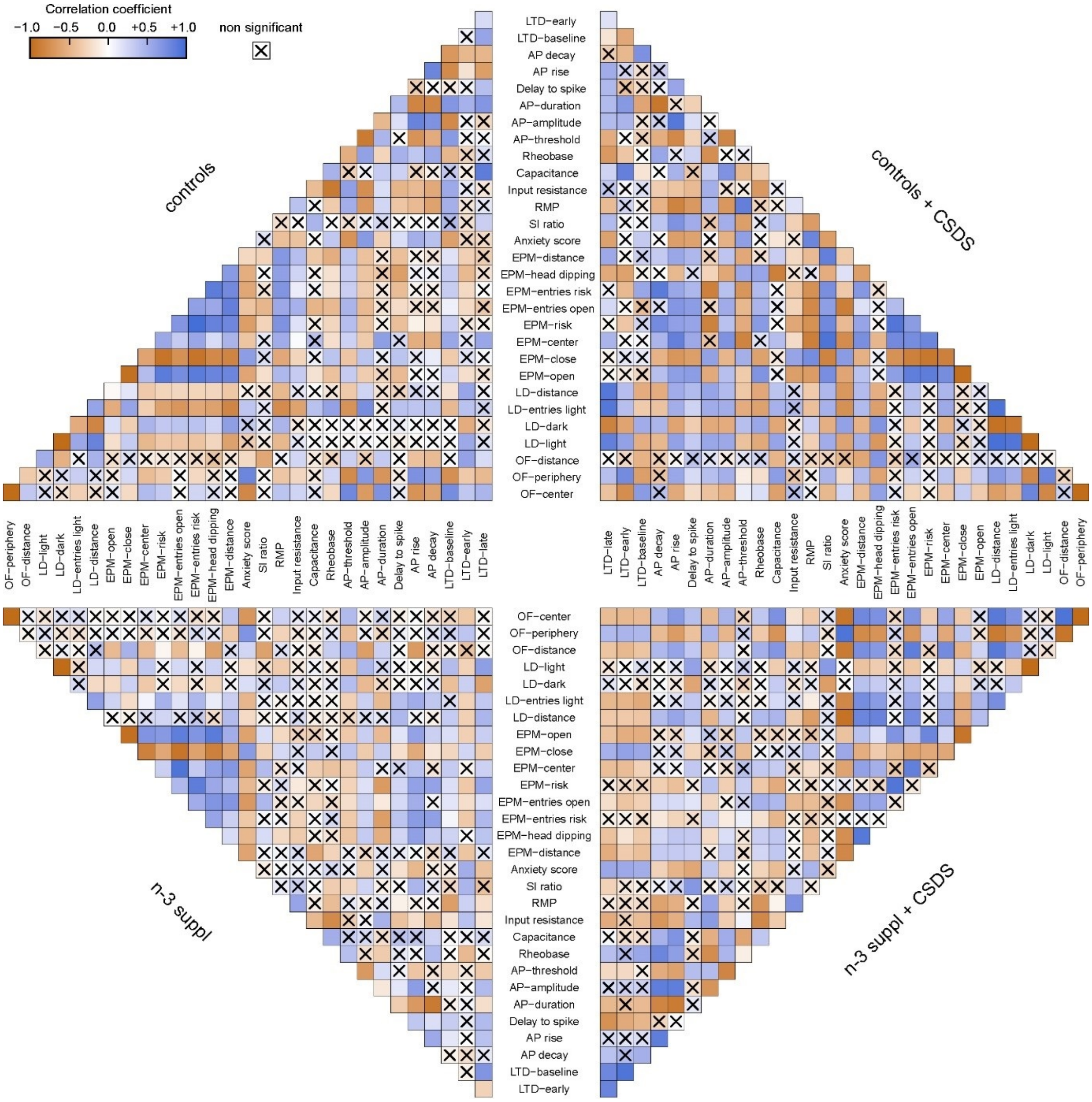

Figure S3. Correlation matrices between behavioral parameters of mice and electrophysiological properties of accumbal MSN. AP: action potential, CSDS: chronic social defeat stress, EPM: elevated plus maze test, LD: light-dark test, LTD: long-term depression OF: open field test, RMP: resting membrane potential, SI: social interaction.

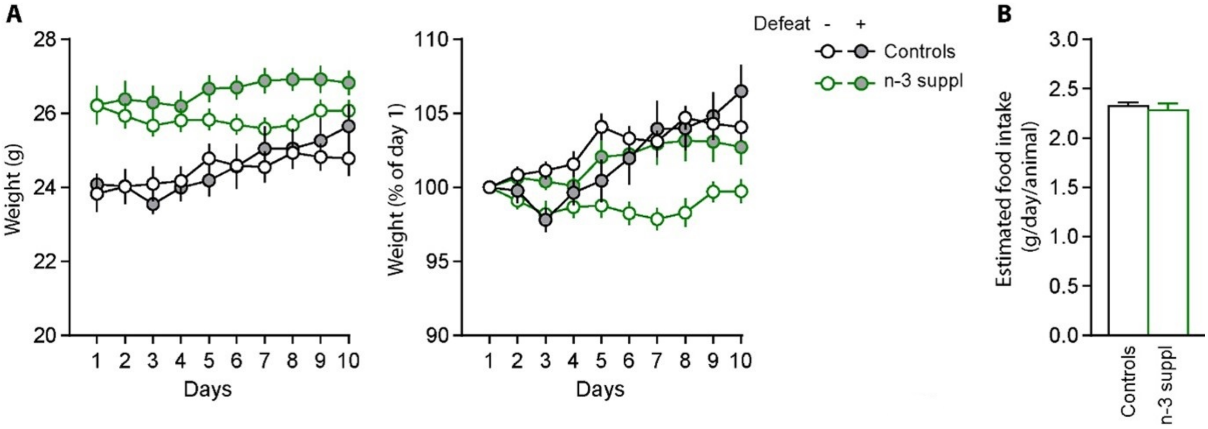

Figure S4. Animal body weight during CSDS and overall food intake. (**A**) Body weight of mice, expressed in g or % of the starting weight (day 1). (**B**) Food intake is similar in both diets (p = 0.61, unpaired t-test). Please refer to Supplemental Table 1 for statistical details.
